# PD45 Changing The Focus: Evaluation Of Ethical, Legal, Social, And Organizational Issues In Health Technology Assessment Reports For Non-Pharmacological Health Technologies

**DOI:** 10.1017/S0266462325101785

**Published:** 2025-12-29

**Authors:** Laura Llinàs-Mallol, Maria Dolors Estrada Sabadell, Joan Segur-Ferrer, Edurne Gallastegui-Calvache, Roland Pastells-Peiró, Rafael Sánchez-Sánchez, Maria Antònia Claveria-Puig, Marc Pellicer-Sarasa, Rosa Maria Vivanco-Hidalgo

## Abstract

**Introduction:**

Ethical, legal, social, and organizational (ELSO) issues are increasingly recognized in health technology assessment (HTA). However, information retrieval methods for ELSO aspects are scarce, and there are no widely accepted standard methods for developing these analyses. Here, we present the methodology and results of the ELSO analysis included in two HTA reports developed in 2024.

**Methods:**

Two HTA reports were developed to analyze the value of: (i) genetic testing in congenital hearing loss (cHL); and (ii) a minimally invasive tissue sampling (MITS) technique to determine cause of death. ELSO domains were considered necessary for decision-making. To address the assessment, we conducted scoping reviews to synthesize the evidence, following the PRISMA Extension for Scoping Reviews, and used a prespecified extraction sheet for defining facilitators and barriers. The literature searches included specific search filters for ELSO issues developed by HTA information specialists from the Spanish Network of Agencies for Health Technology Assessment (RedETS).

**Results:**

The scoping review for ELSO issues in genetic testing for cHL identified 452 references. After eligibility assessment, four reports were included, providing eight ELSO issues: two organizational, two social, and four ethical. Six concerns were facilitators and two were barriers to implementing genetic testing in cHL. The scoping review for ELSO issues in the MITS to determine cause of death identified 903 references. After eligibility assessment, 25 reports were included, bringing forth 43 ELSO concerns: 18 organizational, 15 social, seven ethical, and three legal. Twenty-three issues were facilitators and 20 may be barriers to implementing MITS.

**Conclusions:**

The analysis of ELSO domains through a scoping review provided crucial information for implementing the two selected technologies. However, challenges remain in ELSO information retrieval: (i) key concepts related to ELSO concerns could be described in a variety of ways; and (ii) ELSO terms are not being used or indexed consistently.

